# Neutrophils restricted contribution of *CCRL2* genetic variants to COVID-19 severity

**DOI:** 10.1016/j.heliyon.2024.e41267

**Published:** 2024-12-17

**Authors:** Mattia Laffranchi, Elvezia Maria Paraboschi, Francisco Bianchetto-Aguilera, Nicola Tamassia, Sara Gasperini, Elisa Gardiman, Arianna Piserà, Annalisa Del Prete, Pietro Invernizzi, Angela Gismondi, Alberto Mantovani, Marco A. Cassatella, Rosanna Asselta, Silvano Sozzani

**Affiliations:** a Department of Molecular Medicine, Sapienza University of Rome, Rome, Italy; b Istituto Pasteur-Fondazione Cenci Bolognetti, Rome, Italy; c Department of Biomedical Sciences, Humanitas University, Pieve Emanuele, Milan, Italy; d IRCCS Humanitas Research Hospital, Rozzano, Milan, Italy; e Department of Medicine, Section of General Pathology, University of Verona, 37134, Verona, Italy; f Department of Molecular and Translational Medicine, University of Brescia, Brescia, Italy; g Division of Gastroenterology, Center for Autoimmune Liver Diseases, European Reference Network on Hepatological Diseases (ERN RARE-LIVER), IRCCS Fondazione San Gerardo Dei Tintori, Monza, Italy; h Department of Medicine and Surgery, University of Milano-Bicocca, Monza, Italy; i The William Harvey Research Institute, Queen Mary University of London, London, United Kingdom

**Keywords:** GWAS, COVID-19, ChIP-seq, Neutrophils, SNP, eQTL, Chemokine receptor, CCRL2

## Abstract

The 3p21.31 locus is the most robust genomic region associated with COVID-19 severity. This locus contains a main chemokine receptor (CKR) cluster. We tested expression quantitative trait loci (eQTL) targeting the 3p21.31 CKR cluster linked to COVID-19 hospitalization in Europeans from the COVID-19 HGI meta-analysis. Among these, *CCRL2*, a key regulator of neutrophil trafficking, was targeted by neutrophil-restricted eQTLs. We confirmed these eQTLs in an Italian COVID-19 cohort. Haplotype analysis revealed a link between an increased *CCRL2* expression and COVID-19 severity and hospitalization. By the exposure of neutrophils to a TLR8 ligand, reflecting a viral infection, we revealed specific chromatin domains within the 3p21.31 locus exclusive to neutrophils. In addition, the identified variants mapped within these regions altered the binding motif of neutrophils-expressed transcription factors. These results support that *CCRL2* eQTL variants contribute to the risk of severe COVID-19 by selectively affecting neutrophil functions.

## Abbreviations:

GWAS: genome-wide association study
CKR: chemokine receptor
CCRL2: C-C chemokine receptor-like 2
ACKR: atypical chemokine receptors

## 1 Introduction

The wide heterogeneity that describes SARS-CoV-2 infection and COVID-19 disease cannot be fully explained by host intrinsic factors (age, sex, and comorbidities) [1], and a major role is played by inherited genetic factors. In this respect, germline rare mutations in the type I interferon response genes may be associated with severe forms of COVID-19 [[2], [3]]. In addition, numerous genome-wide association studies (GWAS) identified several genomic loci associated with both COVID-19 severity and susceptibility [[4], [5], [6], [7]]. These findings have been corroborated and expanded through meta-analyses, such as those performed by the collaborative global effort known as COVID-19 Host Genetics Initiative (COVID-19 HGI) [6,8,9]. From the COVID-19 HGI analysis the strongest and most replicated genomic locus associated with COVID-19 severity is situated at 3p21.31^8^. However, due to the complex linkage disequilibrium (LD) structure of the region and its elevated gene density, further analyses are needed to better resolve the association signals in this region [8].

The 3p21.31 locus encompasses several protein-coding genes, including the Chr3 chemokine receptor (CKR) cluster that consists of the *CCR9*, *CXCR6*, *XCR1*, *CCR3*, *CCR1*, *CCR2*, *CCR5*, and *CCRL2* genes. CKRs are involved in cell chemotaxis and orchestrate the immune response by regulating the trafficking of leukocytes to tissues [10]. The roles of *CCR1*, *CCR2*, *CCR3*, CCR5, *CCR9*, *XCR1*, and *CXCR6* in COVID-19 severity have already been described [4,[11], [12], [13]], whereas limited or no information is available regarding *CCRL2*. The *CCRL2* gene codes for C-C chemokine receptor-like 2, a class-A G protein-coupled receptors (GPCRs) structurally related to the atypical chemokine receptor (ACKR) family, a small subclass of CKRs. CCRL2 binds chemerin, a non-chemokine chemotactic protein. Notably, CCRL2 does not signal or internalize upon chemerin binding, nevertheless it regulates leukocyte recruitment in different inflammatory conditions [14,15]. Indeed, we reported the role of Ccrl2 in the fine-tuning of neutrophil migration via the formation of Ccrl2/Cxcr2 heterodimers in mouse neutrophils [16]. Similarly, Ccrl2 was implicated in lung immune surveillance mediated by NK cells [15,17]. In humans it has been reported that *CCRL2* polymorphisms are associated with AIDS progression, and further analysis suggested the role of the *CCRL2* p.Y167F missense variant in *Pneumocystis* pneumonia (PCP) infections [18].

Since the dysregulated lung infiltration by leukocytes is a hallmark of severe COVID-19 [19], the investigation of the CKRs expression within these immune populations is extremely relevant. It has been reported that COVID-19 risk variants may exert their effects in a cell-type-specific manner, mainly through their influence on the regulation of gene expression. For instance, within the 3p21.31 locus, a roaster of severe COVID-19 expression quantitative trait loci (eQTL) variants has been mapped within genome regulatory elements specific to myeloid cells. These variants were associated with an altered expression of *CCR1*, *CCR2*, and *CCR5* in monocytes and macrophages [13]. Moreover, *CCR1* was reported to be expressed by CD16^+^ monocytes, while *CCR5*, *CCR9*, and *CXCR6* by T cell subsets in COVID-19 patients [11,20]. In addition, the expression of *CCR1* and *CCR2* was reduced in peripheral blood monocytes from COVID-19-recovered patients [21].

Severe COVID-19 patients have a limited antiviral response due to local neutrophilia and exacerbated inflammation [[22], [23], [24]]. Given that the role of neutrophils in COVID-19 severity has been extensively described [[25], [26], [27], [28]], but little is still known on the impact of the 3p21.31 locus on neutrophils, we decided to investigate this matter. To this aim, we examined the latest COVID-19 HGI release pertaining COVID-19 hospitalization in individuals of European ancestry. Our analysis identified multiple neutrophils-restricted genetic variants targeting *CCRL2*, which were confirmed in our Italian cohort of COVID-19 hospitalized patients. By stimulating neutrophils with a TLR8 agonist, we mapped neutrophils’ restricted active region targeted by the identified CCRL2 variants. Overall, we propose *CCRL2* as a novel susceptibility factor for COVID-19 severity and hospitalization.

## 2 Results

### 2.1 *CCRL2* eQTLs are novel candidate variants for COVID-19 severity in the European population

**Fig. 1:**
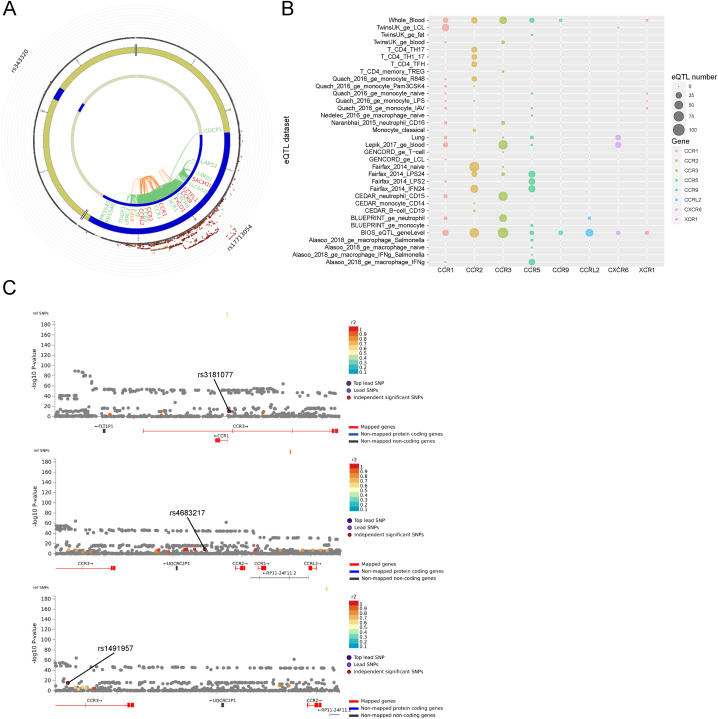
*CCRL2* eQTLs are linked to COVID-19 risk and severity. A) Circos plot of chromatin interactions and eQTLs derived from the A2 COVID-19-hg GWAS meta-analyses round 7 of the European population. Circos plot inner layer is the chromosome ring. Circos plot middle layer is again the chromosome ring where genomic risk loci are highlighted in blue. Circos plot outer layer is the Manhattan plot. Only mapped genes by either chromatin interaction and/or eQTLs are displayed. If the gene is mapped only by chromatin interactions or only by eQTLs, it is colored orange or green, respectively. When the gene is mapped by both, it is colored red B) Bubble plot of the relative *CCRL2* eQTLs divided per eQTL dataset involving immune cells. C) Regional plots of the relative GWAS association p-value for *CCRL2* eQTLs from the A2 COVID-19-hg GWAS meta-analyses round 7 of the European population that acts as eQTLs in neutrophils.

To better understand the 3p21.31 locus impact on COVID-19 severity in neutrophils, we initially explored data from a GWAS meta-analysis of COVID-19 hospitalization in individuals of European ancestry (Cohort A2, freeze 7, COVID-19 HGI European ancestry participants) [8]. This meta-analysis was based on a total of 13,769 severe COVID-19 cases and 1,072,442 healthy controls from the general population. We utilized the Functional Mapping and Annotation (FUMA) [29] resource, which resulted in a fine mapping of the 3p21.31 locus (Fig. 1A; Suppl. Fig. 1). Within this region, we identified a total of 600 SNPs that are mapped to the CKRs gene cluster within a maximum range of 10 kb from the gene body (3:45,927,996–46,454,488, hg19; Suppl. Table 1). Notably, in contrast to earlier releases of the COVID-19 HGI dataset [30], we identified significantly associated SNPs also in proximity of the most distant gene of the CKRs cluster, i.e. *CCRL2* (for a total of 45 SNPs in the gene body; Suppl. Table 1). Hence, we focused specifically on this gene.

Considering that *CCRL2* is predominantly expressed by leukocytes, and in these cells one of the roles of *CCRL2* is to regulate the activities of the co-expressed CKRs [14,16], we identified additional 302 *CCRL2* eQTLs deriving from studies dedicated to COVID-19 relevant tissues (peripheral blood and lungs) (Fig. 1B; Suppl. Table 2). While no eQTLs were identified in the lung catalogue, the majority of eQTLs were derived from the cell-specific BLUEPRINT Neutrophils dataset [31] (Fig. 1B). Importantly, three eQTL variants (rs3181077, rs4683217, and rs1491957) overlapped with significantly associated SNPs derived from our fine-mapping step (Suppl. Table 3). Moreover, additional 11 *CCRL2* neutrophils eQTLs were found in high LD with these three independent significant SNPs (Suppl. Table 3; Fig. 1C).

### 2.2 *CCRL2* eQTL SNPs are associated with COVID-19 severity

The three rs3181077, rs4683217, and rs1491957 eQTLs were further analyzed in an Italian cohort of severe COVID-19 patients and healthy controls [7]. In particular: 527 subjects were affected by severe COVID-19 [median age of 61 ± 8.56 (range: 18–90); 70.4 % males] and 3215 healthy controls from the general Italian population [with median age of 68 ± 12.35 (range: 25–99); 61.9 % males]. Both allelic and genotypic analysis did not highlight any significant association with the disease status (Suppl. Tables 4 and 5). Instead, haplotype analysis evidenced the presence of a trend towards association for the haplotype TCG (P = 0.02, P_permutation = 0.15, Table 1). However, the impact of this SNP combination on gene expression was hard to predict, since the relevant alleles seem to play an opposite role on the expression level of the *CCRL2* gene, based on BLUEPRINT eQTL analysis (Suppl. Table 6).

**Table 1:** Haplotype association analysis for the three selected independent significant SNPs in the Italian population.

Chromosome	Haplotype	Frequency in cases	Frequency in controls	Odds ratio	SNPS	P Value
3	TCG	0.1284	0.1563	0.796	chr3:46209161:C:T|chr3:46233469:C:T|chr3:46331613:G:A	0.02
3	TATGG	0.4962	0.4389	1.33	chr3:46311417:T:A|chr3:46317159:A:G|chr3:46317779:T:G|chr3:46318555:G:A|chr3:46318946:G:A	0.00052

We therefore sought to explore the possible contribution of other relevant eQTLs for the *CCRL2* gene by using the BLUEPRINT database. A total of 3910 eQTLs were present in the neutrophils BLUEPRINT catalogue, 198 of which with a p value < 2∗10^−5^. When we crossed this list with that reporting genome-wide significant SNPs a total of 48 SNPs were in common (Suppl. Table 6). We then performed both allelic and haplotype analyses on these selected SNPs: only two SNPs (rs2213291 and rs4683219) showed a nominal association with the disease at the allelic level (0.0019 and 0.048, respectively; Suppl. Table 6). While rs2213291 stands out as a potential Italian-specific eQTL, as it was not identified individuals of European ancestry (Suppl. Table 2), the rs4683219 variant was already identified in our FUMA fine mapping, being a *CCRL2* eQTL in LD with the already evidenced rs1491957 (Suppl. Table 2).

On the other hand, haplotype analysis disclosed a haplotype associated with the disease (rs62244806, rs113962808, rs2187671, rs34602794 and rs2213291, p = 0.0005, corrected p = 0.07, Table 1). Interestingly, the alleles involved in the haplotype are all associated with an increase of *CCRL2* expression level, according to the eQTL database, thus suggesting that a *CCRL2* level increase may predispose to a severe form of COVID-19 (Suppl. Tables 6 and 7).

### 2.3 *CCRL2* eQTLs are accessible in TLR8-activated neutrophils

To assess the impact of the selected CCRL2 variants on neutrophils, we decided to investigate the active chromatin state of the 3p21.31 locus on neutrophils and monocytes isolated from healthy donors. Neutrophils are known to possess a repertoire of pattern recognition receptors (PRRs), including TLR8, enabling them to detect viral infections by sensing foreign ssRNA [32,33]. Additionally, synthetic TLR8 ligands, such as R848 or GU-rich RNA sequences from the SARS-CoV-2 genome, can profoundly reprogram the transcriptome of human neutrophils [32,33]. Therefore, to confirm the 3p21.31 CKRs cluster gene expression in neutrophils, we utilized previously published data from bulk RNA-seq experiments on ultrapure neutrophils and CD14^+^ monocytes isolated from healthy donors and stimulated with 5 μM R848 for 6 and 20 h^33^.

**Fig. 2:**
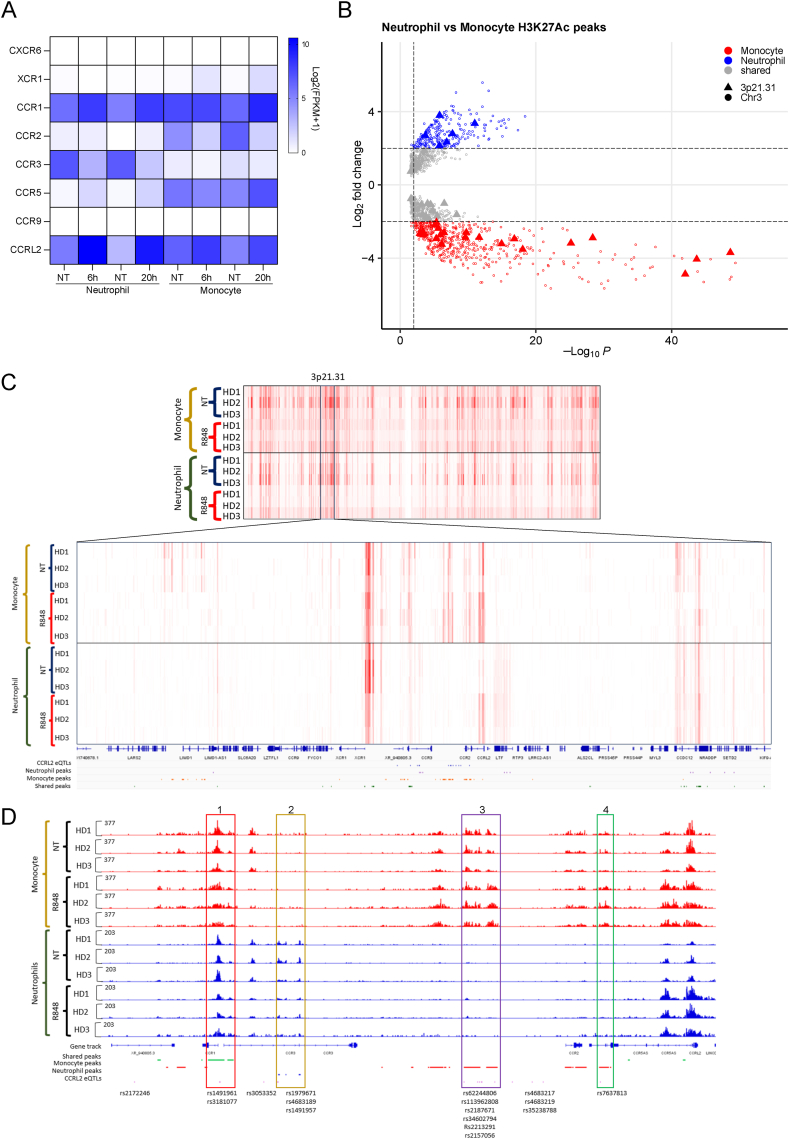
CCRL2 eQTLs are accessible in TLR8-activated neutrophils. A) Heatmap of the expression of the chemokine receptors located within the 3p21.31 locus divided per cell type and treatment. B) Volcano plot of the significantly differentially bound sites between Neutrophils and monocytes sorted by their p-values and log2 of the fold change. The 3p21.31 H3K27Ac significantly enriched peaks are highlighted as triangles. C) Representation of the H3K27Ac levels in the entirety of Chr3 or the 3p21.31 locus of Neutrophils and Monocyte stimulated or not with R848. The green track highlights shared peaks, in orange are showed the Monocyte specific peaks and vice versa, in purple the Neutrophils ones. In blue are highlighted the CCRL2 eQTLs candidates. D) Track of the region involving the independent significant SNPs in the HGI cohort A2 that are eQTL of *CCRL2*, and their co-segregate variants that are also *CCRL2* eQTLs (in blue). The track showed the donor specific H3K27Ac levels of Neutrophils and Monocyte stimulated or not with R848. Shared or either monocyte or Neutrophils specific domains are color coded as above. Red box: shared domain. Gold box: PMN specific domain. Purple box: Monocyte specific domain. Green box: Monocyte specific domain.

Gene expression analysis indicates that *CCR1*, *CCR3*, *CCR5*, and *CCRL2* levels are upregulated with a different magnitude in both neutrophils and monocytes (Fig. 2A), while the other 3p21.31 CKRs were not expressed.

Therefore, to functional validate the identified *CCRL2* eQTLs candidates in neutrophils, we evaluated the active chromatin state in proximity of each SNPs in healthy donor neutrophils or monocytes stimulated with 5 μM R848 for 20h (Fig. 2B–C). We selected the H3K27Ac histone modification as marker of chromatin accessibility, and the resulting ChIP-seq and differential peak analysis at the Chr3 indicated an overall difference between the two cell types (Suppl. Table 8). The volcano plot of the peaks enriched in neutrophils compared to monocytes showed that the majority of the 3p21.31 locus identified H3K27Ac peaks are either monocytes specific or shared between them (Fig. 2B). Still, several H3K27Ac peaks mapped within the Chr3 CKRs cluster were exclusive of neutrophils, as highlighted by the heatmap visualization of the H3K27Ac peaks at the 3p21.31 locus (Fig. 2B–C and Suppl. Table 8). Meanwhile, the region surrounding the *CCRL2* eQTLs candidates is organized in four clear differentially active chromatin regions (Region 1–4; Fig. 2D and Suppl. Table 9). Region 1 is both accessible and active with the same pattern in neutrophils and monocytes. Within this boundary, we observe two *CCRL2* eQTLs, i.e. rs3181077 (associated with COVID-19 severity in Europeans) and rs1491961, both located in a peak for H3K27Ac histone modifications. Region 2, instead, shows H3K27Ac marks only in neutrophils (Fig. 2D). Here, we observe 4 *CCRL2* eQTLs candidates, 3 of which (rs1979671, rs4683189, and rs1491957) located within peaks. Vice versa, regions 3 and 4 showed the opposite trend and are accessible only in monocytes. Here, the mapped *CCRL2* eQTLs candidates are in an inaccessible domain for neutrophils (Suppl. Table 9). In addition, the ChIP-seq of H3K27Ac from other myeloid and lymphoid immune lineages confirmed the neutrophils exclusive accessibility of the region 2 (Suppl. Fig. 2).

To conclude, the identified *CCRL2* eQTLs linked to severe COVID-19 are mostly mapped within active chromatin regions of the 3p21.31 locus that are differentially accessible in neutrophils.

### 2.4 Neutrophils restricted effects of CCRL2 COVID-19-risk variants

Based on the obtained ChIP-seq data, we decided to further filter the *CCRL2* eQTLs candidates based on their accessibility in either neutrophils or monocytes after R848 stimulation. We then queried the RegulomeDB database [34] to further investigate the impact of the selected *CCRL2* eQTLs. Most of them showed a low score, thus suggesting a functional impact of the variants (Suppl. Table 9). To further integrate these observations, we investigated both the RegulomeDB and the JASPAR 2022 TFBS [35] in search for putative transcription factors (TFs) binding sites. To be more stringent, we crossed the extracted information with mRNA expression levels of the TFs expressed by neutrophils after R848 stimulation (Suppl. Fig. 3A and Suppl. Table 9).

**Fig. 3:**
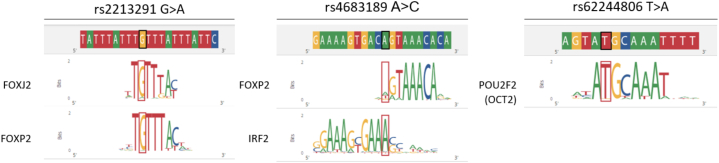
Neutrophils restricted effects of *CCRL2* COVID-19-risk variants. Position weighted matrices (PWMs) representing TFs motifs expressed by Neutrophils that are altered due to the variant rs2213291, rs4683189 and rs62244806 rs2213291.

The rs4683189 polymorphism, which is in LD with the significantly associated rs1491957 variant, is mapped in the neutrophil-exclusive region and it is predicted to impair the binding of the TFs IRF2 and FOXP2, two TFs expressed by neutrophils and actively involved in immune regulatory processes (Fig. 3, Suppl. Fig. 3 and Suppl. Table 9). In addition, two *CCRL2* eQTLs, the “Italian” rs2213291 and the “European” rs62244806, which are mapped in monocyte active regions, are shown to interfere with TFs FOXJ2, FOXP2, and OCT2 (*POU2F2*) (Fig. 3 and Suppl. Table 9). Notably, these TFs are expressed in both neutrophils and monocytes (Suppl. Fig. 3).

We previously reported that the activation of neutrophils via TLR8 promotes a transcriptional OCT2-dependent program. We therefore re-analyzed our previous ChIP-seq experiments of the analytes H3K27Ac, PU.1, and C/EBPb, and OCT2 in R848-treated neutrophils and monocytes, and the overall results supported our observations. In fact, the *CCRL2* eQTLs rs1491957 and rs3181077, which are located in a chromatin region accessible to both neutrophils and monocytes, are mapped within OCT2 and PU.1 peaks. While the variants located in monocyte active regions rs62244806, rs113962808, rs2187671, rs34602794, and rs2213291 are near OCT2 and CEPBβ peaks.

To conclude, the identified *CCRL2* eQTLs linked to severe COVID-19 are mostly mapped within neutrophils' active chromatin regions of 3p21.31, which are targeted by neutrophil-expressed transcription factors.

## 3 Discussion

Neutrophils are a double-edged sword in the context of inflammation and infection. While it is well established their protective role against infections such as from SARS-CoV-2 [36], the impact of genetic variants on neutrophil functions and the host risk to infections remains largely unknown. Despite that, neutrophils hyperactivation can contribute to severe COVID-19 and the related acute respiratory distress syndrome (ARDS) and thrombotic disorders in both children and adults [[37], [38], [39]].

To better understand the role of neutrophils in controlling infections, here we present a fine mapping of the 3p21.31 locus based on the COVID-HGI meta-analysis of individuals of European ancestry. We identified several significantly associated SNPs to severe COVID-19 in proximity of the most distant gene of the chromosome 3 CKR cluster *CCRL2*. Since most of the identified variants were in non-coding regions, and a substantial portion of *CCRL2* eQTLs were predicted to be active in neutrophils, we decided to further investigate these *CCRL2* eQTLs for three main reasons. First, there is still a substantial lack of neutrophils specific genomic studies, mainly due to the several technical difficulties associated to their handling. Second, we have previously reported the role of *CCRL2* in the fine-tuning of neutrophil migration [14]. Third, we and others have identified *CCRL2* as a gene upregulated by pro-inflammatory stimuli in cells types relevant to COVID-19 disease, such as monocytes, neutrophils and endothelial cells [15,33,37,40]. Among the *CCRL2* eQTLs identified, three of them were independent significant SNPs in the COVID-19 HGI cohort, thus suggesting a direct association of *CCRL2* to COVID-19 severity in European ancestry individuals. This association was corroborated by the identification of two haplotypes that link *CCRL2* expression levels to severe form of COVID-19 in an Italian cohort of hospitalized severe COVID-19 patients.

We previously reported that CCRL2 can constitutively form heterodimers with CXCR2, one of the principal neutrophils chemiotactic receptor, and this interaction potentiate the CXCR2 downstream signalling [14]. Therefore, an overall increase in neutrophils CCRL2 expression could significantly impair the proper CXCR2-mediated neutrophil trafficking in COVID-19 patients, thus resulting in a neutrophil hyperactivation state and subsequent severe COVID-19. In fact, *CCRL2* and *CXCR2* expression levels were upregulated in circulating neutrophils from COVID-19 patients [37,41]. In addition, the infiltrating neutrophil-to-lymphocyte ratio is a prognostic value in COVID-19 [42].

Given the close relationship between CCRL2 and neutrophils, we decided to verify the neutrophil-restricted effect of the selected *CCRL2* genetic variants by the assessment of the active chromatin state of the 3p21.31 locus in neutrophils and monocytes incubated with R848, a TLR7/8 agonist [32]. The treatment was able to open chromatin domains within the investigated region, with different availability between neutrophils and monocytes. Still, in all of them, we identified *CCRL2* eQTL candidates. The 3p21.31 locus is a gene-crowded region, with several CKRs whose expression are shared by both neutrophils and monocytes (*CCRL2* included), as demonstrated by our bulk RNA-seq data. Still, we identified a region exclusively utilized by neutrophils. This region is not accessible in other immune cell populations, but it cannot be excluded that other non-hematopoietic cells may have access to it. On the other hand, the identified monocyte-only active domains were independently reported in a previous study, where the authors pinpointed monocyte/macrophage cell-specific accessible chromatin regions in the 3p21.31 locus associated with COVID susceptibility [13]. Yet, they did not find associations for *CCRL2* in both monocytes and macrophages [13], and these observations further strengthen the neutrophil-restricted impact of the selected *CCRL2* variants.

The *CCRL2* targeting genetic variants mapped within neutrophil-active chromatin regions are targeted by neutrophil-expressed transcription factors, such as OCT2, PU.1, CEPB-β, IRF2, and members of the FOX family. While for FOXP2, little is known about its role in neutrophils, FOXJ2 was linked to a different peripheral blood neutrophil count in a GWAS study of hematological traits [43]. Instead, IRF2 is a member of the interferon regulatory transcription factor (IRF) family, and its enhanced activity has been reported in neutrophils from COVID-19 patients [41]. We previously uncovered a transcriptional regulatory circuit active in neutrophils upon TLR8 stimulation that includes interactions between OCT2, PU.1 and CEPB-β, functioning as an amplifier of the transcriptional response induced by proinflammatory stimuli [33]. In that study, we reported that *CCRL2* expression is regulated by this circuit [33]. Collectively these observations point to a functional impact of *CCRL2* eQTLs on regulating its expression levels in neutrophils.

Given the role of *CCRL2* in regulating neutrophil recruitment in inflammatory conditions [14,16], we propose that the effect of the identified CCRL2 genetic variants is not limited to controlling SARS-CoV-2 infections and neutrophil activity. To confirm it, we queried the Open Targets Genetics database [44] for the association of *CCRL2* variants with several haematological traits, including peripheral blood cell counts (Suppl. Table 10). Here, we reported the link between the identified severe COVID-19-linked *CCRL2* variants and the total circulating counts of monocytes, granulocytes, and basophils. These observations further suggest a functional impact of *CCRL2* expression levels in regulating myeloid cell trafficking, and this can be further associated with an increased risk of developing lung disease. In fact, it has been reported that during an RSV (respiratory syncytial virus) infection, lung neutrophils are characterized by upregulation of *CCRL2* [45]. Moreover, a previous report linked *CCRL2* point mutations with AIDS progression and *Pneumocystis pneumonia* (PCP) infections [18]. In tumors, CCRL2 is actively involved in the NK-mediated lung immune surveillance in mouse models of lung cancer [15,17] and has been also reported to be a novel marker for aged TAN (tumor-associated neutrophils) from NSCLC (non-small cell lung cancer) patients [46]. To sum up, our findings on the predisposition of *CCRL2* eQTLs to severe COVID-19 have broader implications for understanding the role of CCRL2 in controlling infections and cancer by finely regulating neutrophil recruitment.

## 4 Limitations of this study

This study focused on the analysis of individuals only of European ancestry, therefore the conclusion obtained may not be universally applicable to other populations. In addition, our genomic analysis focused mainly on the genetic risk to severe COVID-19, and it did not consider other confounding factors such as accessibility to hospitals and socio-economic status.

We centered our analysis on immune cells of the myeloid branch, specifically neutrophils, where we identified several *CCRL2* eQTLs. This does not exclude the role of these variants in non-hematopoietic cells, such as endothelial and other barrier cells, that may express *CCRL2* [16,41]. Investigations on the role of these cells was out of the scope of this manuscript. It is important to note that this manuscript did not utilize a direct model of SARS-CoV-2 infection or had access to neutrophils derived from COVID-19 patients. However, we recently published that single-strand RNAs (ssRNA) from the SARS-CoV-2 genome exhibit a strong capability to activate both human dendritic cells and neutrophils via TLR7/8, with a potency that resembles that of the TLR8 agonist R848 [32,47]. We acknowledge that a viral infection is a complex system involving multiple inflammatory cytokines and various immune cell types [48,49], and our analysis is focused on a confined part of it. Nonetheless, our experiments using R848 to stimulate neutrophils and monocytes serve as an acceptable surrogate for SARS-CoV-2 infection.

Finally, due to technical challenges in handling neutrophils, primarily because of their limited lifespan, it is difficult to further investigate the impact of the identified *CCRL2* eQTLs through dedicated genome editing. Future studies may, therefore, utilize neutrophils from healthy donors who are carriers of *CCRL2* variants to functionally assess their impact on neutrophil biology.

## 5 Materials and methods

### 5.1 COVID-19 GWAS meta-analysis data retrieval

Version 7 (April 8, 2022) COVID-19 GWAS meta-analysis data was retrieved from the COVID-19 Host Genetics Initiative (COVID-19 HGI) [30] at https://www. covid 19 hg.org/. GWAS data (GRCh37/hg19) was obtained from the cohort “A2_ALL_eur_leave_23andme”, involving only Europeans individuals (total of 13,769 very severe respiratory confirmed COVID-19 patients vs 1,072,442 control subjects). GWAS summary statistics files were used to generate input files for FUMA [29] using R.

### 5.2 Fine mapping of COVID-19 associated SNPs within the Chr3 chemokine receptor cluster and eQTL analysis

FUMA [29] was used to analyze the aforementioned A2_ALL_eur GWAS summary with the r2 (LD) parameter set to ≥0.6. Manhattan, regional, and circos plots were generated by FUMA. Chromatin interaction analysis (using the dataset FANTOM5 [50]) as well as eQTL analysis (using datasets relevant for immune cells and COVID-19 pathophysiology, i.e., whole blood and lung, Suppl. Table 2) were performed using FUMA. Thresholds for statistical significance were set to false discovery rate (FDR) < 0.05 and a GWAS p-val < 5 × 10^−8^.

### 5.3 Association and haplotype analysis on the Italian cohort

The Italian cohort comprises 527 unvaccinated subjects affected by a severe form of COVID-19 (all recruited in the period March–May 2020) and 3215 unvaccinated subjects derived from the general population. We considered as severe form of COVID-19 patients hospitalized (in intensive care units and/or general wards) with respiratory failure and a confirmed SARS-CoV-2 viral RNA polymerase-chain-reaction (PCR) test from nasopharyngeal swabs [7]. Respiratory failure was defined according to the highest level of respiratory support required during hospitalization, encompassing supplemental oxygen therapy, non-invasive ventilation, invasive mechanical ventilation, and extracorporeal membrane oxygenation. Details on DNA extraction, genotyping, QC, and imputation procedures have already been reported [7]. Allelic and genotypic association tests were performed using the PLINK v.1.9 (https://www.cog-genomics.org/plink/) logistic regression framework, introducing age, sex, age × age, sex × age, and the first ten principal components as covariates. Analyses were conducted always referring to the minor allele.

Power estimates indicate that a polymorphism (20 % frequency, like the rs3181077) were to confer a 1.5-fold increase in COVID-19 susceptibility, 500 cases vs 3200 controls would be of sufficient size to have 82 % power to detect a significant association at P = 0.001.

Beagle v3.3 (http://faculty.washington.edu/browning/beagle/b3.html) was used to infer haplotype phases and to perform haplotype association analysis. In this case, the default setting of 1000 permutations was used to calculate corrected P values, with a significant threshold settled to 0.05. PLINK v.1.07 (https://zzz.bwh.harvard.edu/plink/) was used to calculate haplotype frequencies.

### 5.4 eQTL retrieval from BLUEPRINT database

For *CCRL2* eQTL analysis, we retrieved data from the EBI database (https://www.ebi.ac.uk/eqtl/Data_access/) specifically on neutrophils (study ID: QTS000002, dataset ID: QTD000026, tissue ID: CL_0000775). Only those eQTLs localized within 500 kb around the gene were considered. A p-val < 2 × 10^−5^ was set as a threshold for significance, and the variants selected have a p-val < 5 × 10^−8^ in the COVID-19 HGI cohort.

## 6 Cell purification and culture

Granulocytes were isolated from buffy coats of healthy donors and manipulated under endotoxin-free conditions. After Ficoll-Paque gradient centrifugation, followed by dextran sedimentation and hypotonic lysis of erythrocytes, neutrophils were isolated to reach 99.7 ± 0.2 % purity, by positively removing any eventual contaminating cells using the EasySep neutrophil enrichment kit (StemCell Technology, Vancouver, Canada) [51]. Human CD14+-monocytes were isolated from PBMCs by anti-CD14 microbeads (Miltenyi Biotec, Bergisch Gladbach, Germany) to reach >98 % purity. Neutrophils and monocytes were then suspended at 5 × 10^6^/ml and 3 × 10^6^/ml, respectively, in RPMI 1640 medium supplemented with 10 % low endotoxin FBS (<0.5 EU/ml; from Sigma, Saint Louis, MO, USA), treated or not with 5 μM R848 (InvivoGen, San Diego, CA, USA), seated either in 6/24-well tissue culture plates or polystyrene flasks (Greiner Bio-One, Kremsmüster, Austria) and cultured at 37°, 5 % CO2 atmosphere. After the desired incubation period, cells were processed for ChIP-experiments.

### 6.1 Chromatin immunoprecipitation (ChIP) sequencing (ChIP-Seq)

Protein-DNA cross-linking was achieved by incubating 2.5 × 10^6^ neutrophils or monocytes with 1 % formaldehyde for 10 min at room T, under gentle agitation. Cross-linking reaction was stopped by adding glycine to a final concentration of 125 mM, and incubating cells at room T for five more minutes. After fixation, cells were washed with ice-cold PBS, collected by scraping, and finally pelleted by centrifugation (5 min, 300×*g*, 4 °C). Pellets were suspended in 900 μL L1 lysis buffer (50 mM Tris, pH 8.0, 2 mM EDTA, 0.1 % IGEPAL, 10 % glycerol) containing protease inhibitors. Nuclei were pelleted at 1000×*g* at 4 °C and resuspended in 300 μL L2 lysis buffer (50 mM Tris, pH 8.0, 1 % SDS, 5 mM EDTA) including protease inhibitors. Chromatin was sheared to an average DNA size of 300–400 bp by sonication on wet ice [6 pulses of 15 s at the 50 % maximum potency, with 15 s pauses, using a BANDELIN SONOPLUS ultrasonic homogenizers HD 2070 (Bandelin, Berlin, Germany)]. Lysates were then cleared by centrifugation to remove debris (10 min, 13000×*g*, 12 °C), and diluted 10x in dilution buffer (50 mM Tris, 5 mM EDTA, 200 mM, 0.5 % IGEPAL). Immunoprecipitations were carried out overnight at 4 °C using 5 μg/ml antibodies. Immune complexes were then collected by adding 15 μl of Dynabeads Protein A (Thermo Fisher Scientific, Waltham, MA, USA) for 1 h at 4 °C under gentle rotation. Beads were then immobilized on a magnetic support and washed three times in washing buffer (20 mM Tris, pH 8.0, 0.1 % SDS, 2 mM EDTA, 1 % IGEPAL, 500 mM NaCl) and once in TE. The resulting protein complexes were then eluted in TE containing 2 % SDS and reversed crosslinked by overnight incubation at 65 °C. Antibodies toward H3K27Ac (ab4729) were from Abcam. The DNA was purified by QiaQuick PCR purification kit (QIAGEN) according to the manufacturer’s instructions and eluted in 50-100 μl. ChIP DNA was prepared for sequencing following TruSeq DNA sample preparation guide (Illumina, Cambridge, UK). In brief, 10–50 ng purified DNA from chromatin immunoprecipitation, obtained from different amounts of cells according to the antibody used for the ChIP, were adaptor-ligated and PCR-amplified according to the manufacturer’s protocol (Illumina). Sequencing libraries were multiplexed and ran on Illumina sequencer. Finally, reads were quality-filtered according to the Illumina pipeline [52].

### 6.2 RNA-seq computational analysis

Computational analysis of transcriptome datasets generated by Smart-seq2 has been performed using the following bioinformatic pipeline. FASTQ files deriving from the published RNA-seq dataset GSE123532 were filtered according to the Illumina pipeline, and the contaminant adapters in the FastQ files (single-end 75 bp) were detected using FastQC v0.11.8. Then, adapters and base quality trimming were performed using Trim Galore (https://www.bioinformatics.babraham.ac.uk/projects/trim_galore/)! script with parameters -length 50. To improve the quality of the mapping, reads were further trimmed at the 3′ to a length lower than 72 bp being discarded. Trimmed reads were quantified using Kallisto quant (Bray et al., 2016) to the human reference transcriptome GRCh38v96 obtained from ENSEMBL web site (www.ensembl.org/index.html) and applying parameters –bias –single -l 200 -s 20 –genomebam. Kallisto performs transcript level quantification estimated from Smart-seq2; transcripts were combined to gene level using tximport packages. Gene counts were normalized among various samples using DESeq2, and only genes coding to protein and long non-coding RNA (lnRNA) were retained for downstream analysis. DESeq2 was used to generate the expression metric and fragment per kilobase of transcript per million mapped reads (FPKM). FPKM normalization divides the read count for each gene by the length of the transcript for that gene, and then, scales all read counts per million reads in the data file. This normalization step allows comparison of expression levels between two genes in the same sample, or of the same gene between different samples. To avoid possible noise of genes expressed at very low levels, only genes expressed above 1 FPKM in at least one sample were considered as “expressed” genes and retained for downstream analysis. Gene expression FPKMs were log2-transformed.

### 6.3 ChIP-seq bioinformatic analysis

Raw sequencing output BCL data were converted to FASTQ files by using the Illumina pipeline software bcl2fastq v2.20. The quality of the reads was checked by FastQC v.0.11.8 (https://www.bioinformatics.babraham.ac.uk/projects/fastqc/). Adaptors and low-quality reads were trimmed by Trim Galore v0.63 using default parameters. To avoid effects derived from different reads lengths, reads were truncated to 51bp using fastx_trimmer from the FASTX-toolkit (http://hannonlab.cshl.edu/fastx_toolkit/). Trimmed reads were mapped to the human genome (Genome Reference Consortium GRCh38, Dec/2013) using Bowtie2 v2.3.5.1 with default parameters (Langmead et al., 2009), and deduplication was performed with markdup of Sambamba v0.6.7. Only reads mapping to the nuclear genome were kept for downstream analyses. Tracks were generated and were linearly rescaled to the same sequencing depth (10 million of mapped reads), by using HOMER analysis package.

## 7 Identification of H3K27Ac-enriched regions by ChIP-seq

The identification of peaks for H3K27Ac and TFs, such as OCT2, PU.1 and CEBPB, was carried out by MACS3 (v3.00b3) [53] with parameters set to --gsize hs --broad --broad-cutoff 0.1 –nomodel --extsize 146, while --gsize hs and default setting was applied for TFs. Peaks overlapping ENCODE blacklist regions were removed and biological replicates were merged using MSPC (v6.0.0) [54] to generate consensus peaks. Consensus H3K27Ac coordinates were re-centered to the best nucleosome free regions (NFR) within a 200 bp window, using the command “getPeakTags –nfr” from HOMER package, and then resized to 2 kb as performed by Heinz et al., 2013 [53]. TFs and histone modification regions were assigned to genes with the “nearest TSS” criteria. Annotated positions for promoters, exons, introns and other genomic features were based on Human transcriptome annotation (GRCh38v96 ENSEMBL).

### 7.1 Differential peak analysis

Differential H3K27ac occupancy and TFs binding (|log2[fold change]| > 2) and adjusted p-value <0.05 was performed using Bioconductor/R “DiffBind” v2.14 package (https://bioconductor.org/packages/release/bioc/html/DiffBind.html). The corresponding ChIP-seq BAM files were used as the input data for the analysis. Briefly, overlaps of peaks were examined to determine how well similar samples cluster together with the function dba.count. Second overlaps reads in each cell type and condition for each sample were counted with the function dba.contrast. Third, the core analysis of DiffBind was executed by default using DESeq with the function dba.analyze adding as interactions the R848 treatment. Finally, the results were reported the function dba.report. The significant peaks (p-value <0.05) changing less than 2 fold change under the two conditions were defined as “common” (Heinz et al., 2010). Resulting plots were obtained using DiffBind graphical options or with the Bioconductor/R package “EnhancedVolcano” (v3.18).

### 7.2 Functional characterization of identified candidate variants

The selected *CCRL2* eQTLs were probed for their pathogenicity with RegulomeDB [34] (v2), thus obtaining the dedicated risk scores and positional weight matrices (PWM) for the putative transcription factor binding. The PWM matrix was derived from the JASPAR 2020 CORE collection [55]. We selected only those transcription factors which were expressed by Neutrophils (FPMK counts ≤ 5) with or without the R848 treatment.

Peak calling files of H3K27Ac, PU.1, CEBPβ and OCT2 ChIP-seq from *in vitro* stimulated human neutrophils were obtained from GSE119395.

### 7.3 Software availability

The software utilized in these analyses were: R (v4.0.2), Rstudio (v4.1.5), the packages: DESeq2 (v1.20.0) DiffBind (v2.14), EnhancedVolcano (v3.18). MACS3 (v3.0.1), HOMER (v4.11), bcl2fastq (v2.20), FastQC (v.0.11.8), Trim Galore (v0.63), FASTX-toolkit (v0.0.13), Bowtie2 (v2.3.5.1), Sambamba (v0.6.7) and Prism (v7.04). Data were visualized with IGV (Integrative Genomics Viewer, https://doi.org/10.1093/bioinformatics/btac830).

### 7.4 Statistical analysis

Regarding the ChIP-seq and bulk RNA-seq experiments, statistical evaluation was performed accordingly to the indicated methodologies in the relevant method sections using one-way or two-way analysis of variance, followed by Tukey’s post hoc test. Values of p < 0.05 were considered statistically significant. Data are expressed as means ± SD.

## CRediT authorship contribution statement

**Mattia Laffranchi:** Writing – review & editing, Writing – original draft, Formal analysis, Conceptualization. **Elvezia Maria Paraboschi:** Methodology, Investigation. **Francisco Bianchetto-Aguilera:** Methodology, Investigation. **Nicola Tamassia:** Methodology, Investigation, Funding acquisition. **Sara Gasperini:** Methodology. **Elisa Gardiman:** Methodology. **Arianna Piserà:** Methodology. **Annalisa Del Prete:** Supervision. **Pietro Invernizzi:** Supervision. **Angela Gismondi:** Supervision, Funding acquisition. **Alberto Mantovani:** Supervision. **Marco A. Cassatella:** Supervision, Funding acquisition. **Rosanna Asselta:** Writing – review & editing, Writing – original draft, Supervision, Funding acquisition, Conceptualization. **Silvano Sozzani:** Writing – review & editing, Writing – original draft, Supervision, Funding acquisition, Conceptualization.

## Ethics and consent

This study was approved by: i) Azienda Ospedaliera Universitaria Integrata Verona (Verona, Italy; study ID 5626 of February 02, 2012); ii) Humanitas Clinical and Research Center, IRCCS (reference number, 316/20); and iii) the University of Milano–Bicocca School of Medicine, San Gerardo Hospital, Monza (the ethics committee of the National Institute of Infectious Diseases Lazzarro Spallanzani reference number, 84/2020).

All participants provided written informed consent to participate in the study and for their data to be published. This study did not involve minors.

## Data accessibility

ChIP-seq and RNA-seq data are publicly available at the Gene Expression Omnibus database (https://www.ncbi.nlm.nih.gov/geo/) under the accession numbers GSE119395 and GSE269643.

## Computer code

No unique code was generated in this article.

## Declaration of competing interest

The authors declare that they have no known competing financial interests or personal relationships that could have appeared to influence the work reported in this paper.
