# Supplementary figures and images for: Neutrophils restricted contribution of CCRL2 genetic variants to COVID-19 severity
Source: Heliyon. 2024 Dec 17;11(1):e41267. doi: 10.1016/j.heliyon.2024.e41267 (PMC11731188; doi:10.1016/j.heliyon.2024.e41267)

A

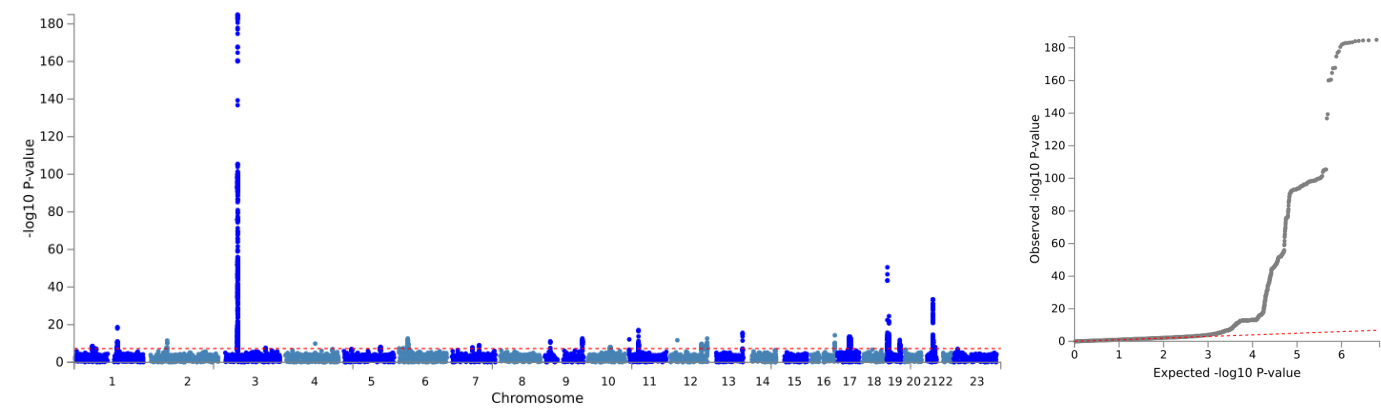

B

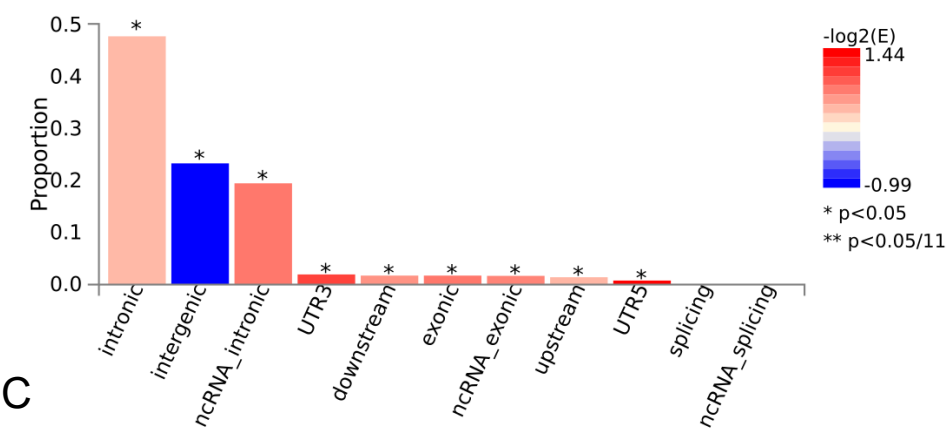

C

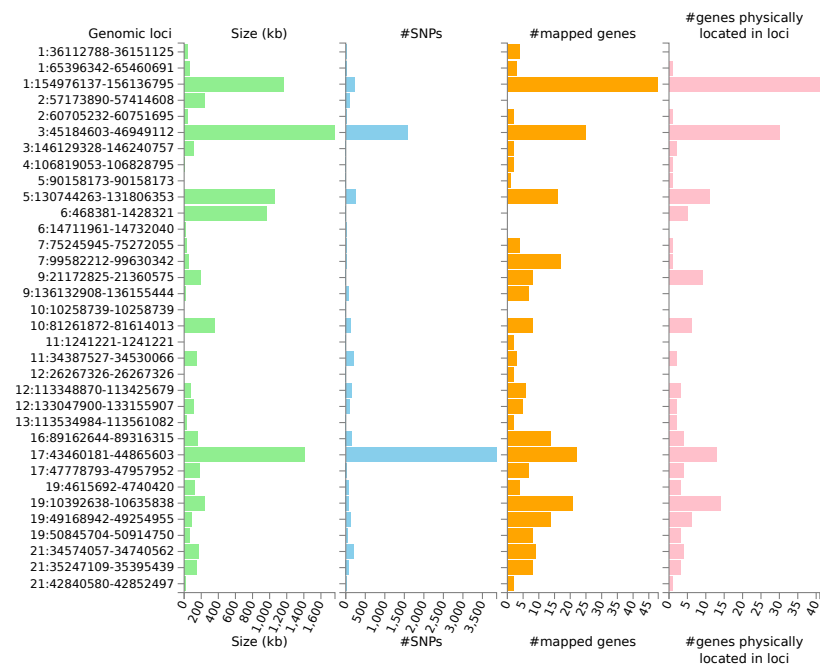

Supplement: Multimedia component 1 [file mmc1.pdf]

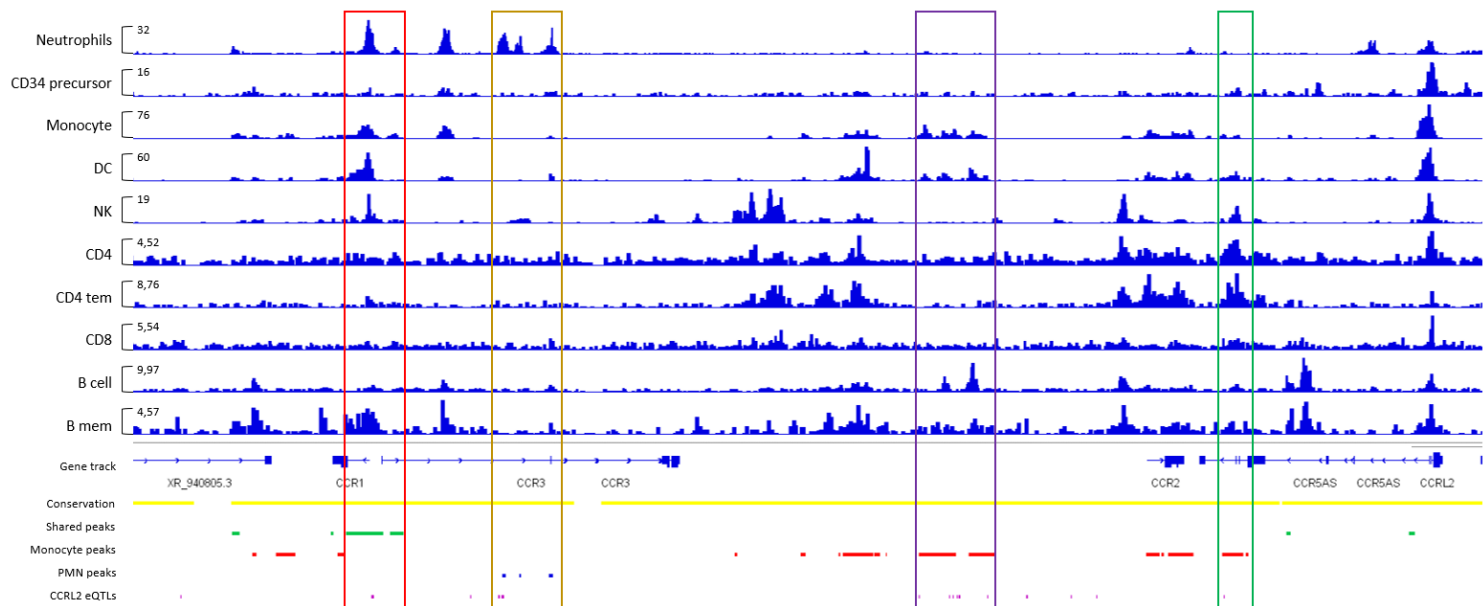

Supplement: Multimedia component 2 [file mmc2.pdf]

A

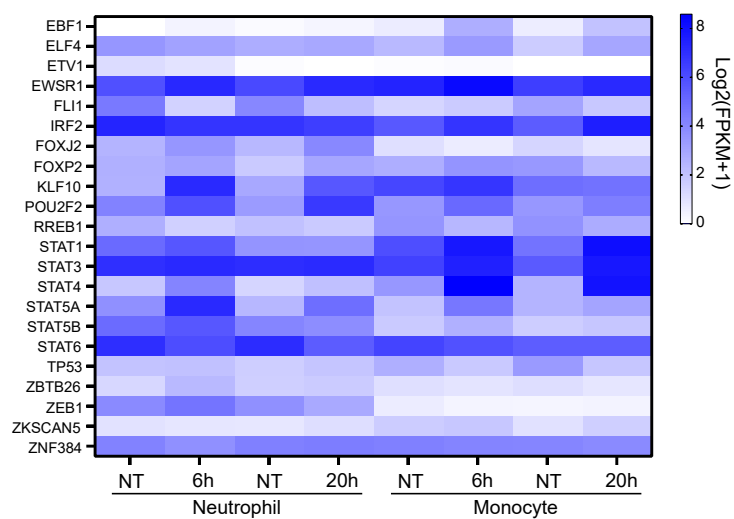

B

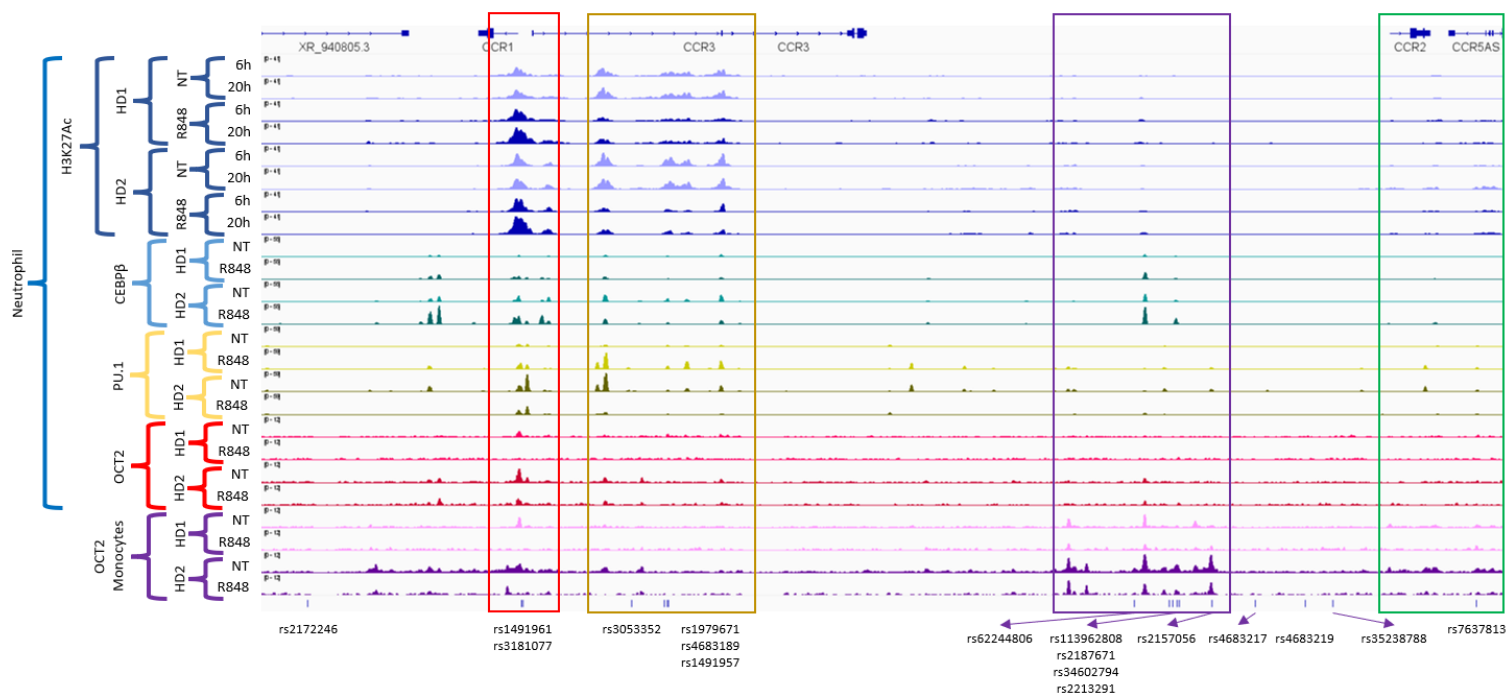

Supplement: Multimedia component 3 [file mmc3.pdf]
